# Detecting spatial clusters of HIV and hepatitis coinfections

**DOI:** 10.1371/journal.pone.0203674

**Published:** 2018-09-18

**Authors:** Suparna Das, Jenevieve Opoku, Adam Allston, Michael Kharfen

**Affiliations:** 1 Strategic Information Division HIV/AIDS, Hepatitis, STD and TB Administration (HAHSTA), District of Columbia Department of Health, Government of the District of Columbia, Washington DC, United States of America; 2 HIV/AIDS, Hepatitis, STD and TB Administration (HAHSTA), District of Columbia Department of Health, Government of the District of Columbia, Washington DC, United States of America; Centers for Disease Control and Prevention, UNITED STATES

## Abstract

**Background:**

People with HIV infection in the United States are often affected by chronic viral hepatitis. These coinfected people with either HBV or HCV are at increased risk for serious, life-threatening complications. Coinfections with viral hepatitis may also complicate the delivery of anti-retroviral therapy (ART) by escalating the risk of drug-related hepatoxicity. According to the Centers for Disease Control and Prevention (CDC), approximately 10 percent of people with HIV in the United States also have HBV, and 25 percent also have HCV coinfection. With the advent of highly active antiretroviral therapy (HAART) and the increased life-expectancy of HIV patients, clinicians are more likely to be confronted with issues related to co-infection and the management challenges that they present, especially in resource-limited settings. The purpose of this analysis was to identify geographical clusters of HIV- (HBV/HCV) co-infection and compared to the geographical clusters of not co-infected using DC, Department of Health surveillance data. The results of the analysis will be used to target resources to areas at risk.

**Methods:**

HIV and Hepatitis surveillance data were matched among cases diagnosed between 1980 and 2016. HIV-hepatitis co-infected and the not co-infected spatial clusters were detected using discrete Poisson model. Kulldorff’s spatial scan statistic method was implemented in the free software tool called SaTScan which has been widely adopted for detecting disease cluster. The analysis was conducted by tracts, but for visualization, ease of interpretation and assist in policy making the tract map was overlaid with the ward map using ArcGIS 10.5.1.

**Results:**

Between 1980 and 2016, there were 12,965 diagnosed cases of HIV, of which 2,316 HIV/Hepatitis matches were identified. Of the 2316 co-infected people living in DC, 25 percent (N = 590) of people had HBV, and 75 percent (N = 1,726) had HCV. Out of 12,965 diagnosed cases, remaining 10,649 did not have any co-infections (not co-infected). IDU (27.16 percent) and MSM (32.86 percent) were the highest mode of transmission for co-infected population. African-American were reported 83.64 percent (N = 1,937) among co-infection population. Three clusters were identified for both co-infected population in DC. The largest cluster radius for co-infected analysis covers wards 6, 7 and 8 as well as large parts of 2 and 5 (*p < 0*.*001*). Multiple clusters were identified for not co-infected population (*p < 0*.*001*). IDU (n = 450) was the highest mode of transmission for the co-infected clusters. For all clusters combined of not co-infected population highest mode of transmission were MSM (n = 2,534). This analysis also showed racial disparity, economic deprivation and lack of education were prominent in the co-infected clusters.

**Conclusion:**

We identified locations of high risk clusters where enhanced hepatitis and HIV prevention, treatment, and care can help combat the epidemic. The clusters radius expands into the neighboring state of Maryland as well. The findings from this analysis will be used to target area based public health policy and healthcare interventions for HIV-hepatitis. It is recommended based on the analysis that needle exchange programs can successfully control new HIV infections as well as hepatitis co-infections.

## Introduction

Viral Hepatitis is a medical condition characterized by inflammation of the liver triggered by a virus. Hepatitis B (HBV) and Hepatitis C (HCV) infections are common among people who are at risk for or living with, HIV. People may get infected with viral hepatitis the same way as HIV—through sexual contact without a condom and sharing needles to inject drugs [1]. HIV and hepatitis (B and C) share common transmission routes, which also include maternal and perinatal[2]. According to the Centers for Disease Control and Prevention (CDC), approximately 10 percent of people with HIV in the United States also have HBV, and 25 percent also have HCV coinfection.

Viral hepatitis causes liver-related health issues among people with HIV (co-infected) more than among those who do not have HIV. Though treatment with antiretroviral therapy (ART) has improved the health and extended the life expectancy of people with HIV, liver disease—much of which is hepatitis related non-AIDS-related deaths is common in this population [1]. Coinfection with viral hepatitis may complicate the delivery of ART by escalating the risk of drug-related hepatoxicity[3]. For these reasons, expert guidelines developed in the United States and Europe recommend screening all HIV-infected persons for co-infection with hepatitis [3].

The estimated prevalence rate of HIV in District of Columbia (DC) being at 1.9 percent exceeds the World Health Organizations definition of 1 percent for generalized epidemics [4]. District residents aged 40 years and over continue to be disproportionately impacted by HIV. Approximately 3.7 percent of residents whose current age is 40 to 49 years and 5.2 percent of residents aged 50 to 59 years living with HIV [4]. With the advent of highly active antiretroviral therapy (HAART) and the increased life-expectancy of HIV patients, clinicians are more likely to be confronted with issues related to co-infection and the management challenges that they present, especially in resource-limited settings [5]. Though HIV and hepatitis coinfections have been studied considerably, [6–9] there is a lack of studies that have identified spatial clusters of co-infections in many parts of the United States that bear substantial co-infection burdens such as DC. The majority of HIV studies have focused on the prevalence of morbidity or premature mortality and often do not take into account the spatial dimension in disease or risk factors of HIV to identify high-risk areas for public health intervention and healthcare intervention programs.

Geographical Information Science (GIS), spatial epidemiological and statistical methods offer a rigorous approach to detect clusters of disease, which can inform public health policy and targeted interventions [10]. Spatial cluster analysis plays a significant part in public health. It can assist in finding areas that have unusually high disease occurrence which in turn helps to evaluate health care availability and health care operations [11]. Confirmed clusters are also useful to define the areas that are in need of further investigation and potential intervention [11]. Spatial cluster may be defined as a collection of neighboring entities that are more alike to each other than to objects external to the cluster [12]. The purpose of the analysis was to identify spatial clusters of HIV–(HBV/HCV) co-infection in District of Columbia (DC) and compare them to the high-risk clusters of people who do not have any hepatitis co-infection (not co-infected).

## Data

HIV and hepatitis surveillance data were matched among cases diagnosed between 1980 and 2016. Between 1980 and 2016, there were 12,965 diagnosed cases of HIV, of which 2,316 HIV/Hepatitis matches were identified. The record linkage was performed here in the surveillance division of the HIV/AIDS, Hepatitis, STD, and TB Administration (HAHSTA), District of Columbia Department of Health (DOH). The records were then aggregated by census tracts of District into counts. Thus the data was de-identified in the process. For this analysis, HIV cases were defined using the CDC 2014 revised classification system of HIV [13]. The outcome variables were coded as ‘co-infected’ = 1 and ‘not co-infected’ = 0. An individual with HIV was categorized as co-infected if he or she had been concomitantly infected with confirmed hepatitis case based on the CDC case definition [14–17] and currently residing in DC according to the last laboratory report. The hepatitis B and hepatitis C co-infections were lumped together for the analysis owing to their similarities in distribution thus avoids redundancy of analysis. The characteristics of HIV-infected and co-infected individuals used in this analysis included sex, age, race, mode of transmission and current HIV care status.

The geographic coordinates associated each case of infection was assigned using Maptitude Geographic Information System software. Postbox numbers which comprised of a negligible percentage of the cases were not included as they cannot be geocoded for the lack of physical addresses. Cases diagnosed at the DC Detention centers were left out of the analysis as they would lead to spatial bias in the analysis. For homeless cases addresses of the shelters would also raise the issue of spatial bias were excluded from the analysis. The geocoded cases were aggregated by census tracts. The shapefile of the census tracts and wards were obtained from Office of Chief Technology Officer (OCTO), Government of District of Columbia. The data was obtained from DC Hepatitis registry and Enhanced HIV/AIDS Reporting System (EHARS) of HAHSTA within the DOH.

## Methods

A map of HIV prevalence by census tracts was created for DC by using information on HIV cases from 1980–2016 (S2 Fig). Descriptive analyses of the HIV and hepatitis were performed in SPSS (IBM Inc., USA).

Kulldorff’s spatial scan statistic method was implemented in the free software tool called SaTScan [18] which has been widely adopted for detecting disease cluster [19,20]. Kulldorff’s spatial scan statistic method places a circular scanning window at each of the point locations within an analysis area. At each of these point locations, the radius of the circle is varied from a size of zero (i.e., covering only a single point) to 1 km radius. In this manner, the method generates a large number of distinct circular windows, each including a different set of neighboring points for the clustering test. The windows that have a significantly high concentration of events are considered to be ‘spatial clusters.’ The null hypothesis of the Kulldorff’s spatial scan statistic states that the event is randomly distributed in geographic space and that the expected event count is proportional to the population at risk [11]. We used purely spatial discrete Poisson spatial analysis for this analysis, and the details of the method and calculation are described in a series of papers [21–25]. A significant p-value was considered to be *<0*.*01*. For the discrete Poisson analysis, a case (co-infected and not co-infected) and a population (total population in each tract) file were created with the tracts as the geographic unit. A coordinates file containing the latitude and longitude at the centroid of each tract was used to define the locations in both analyses.

SaTScan lacks cartographic support for understanding the clusters in a geographic context. Thus the results were exported into ArcGIS version 10.5.1 (Environmental Systems Research Institute, Redlands, CA, USA) and mapped for visualization purposes. There are 179 US census-defined tracts and eight wards in DC (S1 Fig). The analysis was conducted by tracts, but for visualization, ease of interpretation and assist in policy making the tract map was overlaid with the ward shapefile.

## Results

Between 1980 and 2016, there were 12,965 diagnosed cases of HIV, of which 2316 HIV/Hepatitis (co-infected) matches were identified. Of the 2,316 co-infected people living in DC, 25 percent (N = 590) of people had HBV, and 75 percent (N = 1726) had HCV. Out of 12,965 diagnosed cases, remaining 10,649 did not have any co-infections (not co-infected). 26.51 percent of the co-infected population were females, 72.19 percent were males, and 1.30 percent were transgender. 84.64 (N = 1,937) percent of the diagnosed co-infected population were African American followed by white who were 26.51 percent (N = 614). For not co-infected population the percentages were similar with male (72.12 percent), females (26.12 percent) and transgender (1.76 percent) (Table 1). The proportions of racial burden among co-infected and not co-infected people in DC also showed comparable trends. Older population (50–59 and 60+) carried a significant burden of co-infections, for it was not co-infected population (40–49 and 50–59). Men who have sex (MSM) (32.86 percent) and Injection Drug Users (IDU) (27.16 percent) followed by heterosexual contact (23.36 percent) was the largest mode of HIV transmission for the co-infected population. For non-infected population, MSM (46.76 percent) followed by heterosexual contact (28.58 percent) were the largest modes of transmission of HIV. Over the years diagnosis have increased particularly, with the implementation of generalized testing efforts across the jurisdictions.

**Table 1:** Comparative characteristics of HIV and hepatitis B or C coinfected (n = 2316) and not co-infected (n = 10649) of individuals by sex, race, age, year of HIV diagnosis and mode of transmission.

	Co-Infected Population		Not Co-Infected Population	
	Number	Percent	Number	Percent
**Gender**				
Female	614	26.51	2782	26.12
Male	1672	72.19	7680	72.12
Transgender	30	1.3	187	1.76
**Race**				
White	230	9.93	2076	16.01
Black	1937	83.64	9671	74.59
Hispanic	97	4.19	884	6.82
Other	52	2.25	334	2.58
**Age Group**				
> = 60	753	32.51	1515	14.23
13–19	0	0	60	0.56
20–24	7	0.3	324	3.04
25–29	45	1.94	863	8.1
30–39	234	10.1	2218	20.83
40–49	382	16.49	2581	24.24
50–59	894	38.6	3063	28.76
Pediatric	1	0.04	22	0.21
**Year of Diagnosis**				
Years before 1996	546	23.58	1390	13.05
1997 to 2002	605	26.12	1959	18.4
2003 to 2015	1165	50.3	7300	68.55
**Mode of Transmission**				
MSM	761	32.86	4980	46.76
IDU	629	27.16	743	6.98
MSM/IDU	140	6.04	277	2.6
Heterosexual contact	541	23.36	3044	28.58
Risk not identified	232	10.02	1471	13.81
Other	4	0.17	6	0.06
Perinatal	9	0.39	128	1.2
**Total**	2316		10649	

For retained in HIV care and viral suppression status co-infected, and not co-infected population showed comparable proportions (Table 2). Though ever virally suppressed among coinfected population is 80.79 percent compared to 18.02 percent of not co-infected people. The HIV care for both co-infected and not co-infected population did not register any differences based on this descriptive analysis.

**Table 2:** Comparative characteristics of HIV and hepatitis B or C coinfected (n = 2316) and not co-infected (n = 10649) of individuals by care pattern.

	Co-Infected Population		Not Co-Infected Population	
	Number	Percent	Number	Percent
**Retained**				
No labs	730	31.52	3832	35.98
Retained	484	20.9	1919	18.02
Sporadic	1102	47.58	4898	45.99
**In Care**				
No	730	31.52	3832	35.98
Yes	1586	68.48	6817	64.02
**Virally Suppressed**				
Not Suppressed	203	8.77	918	8.62
Suppressed	1334	57.6	5706	53.58
Unknown	779	33.64	4025	37.8
**Ever Virally Suppressed**				
Not Suppressed	416	17.96	3832	35.98
Suppressed	1871	80.79	1919	18.02
Unknown	29	1.25	4898	45.99
**Total**	2316		10649	

**Fig 1:**
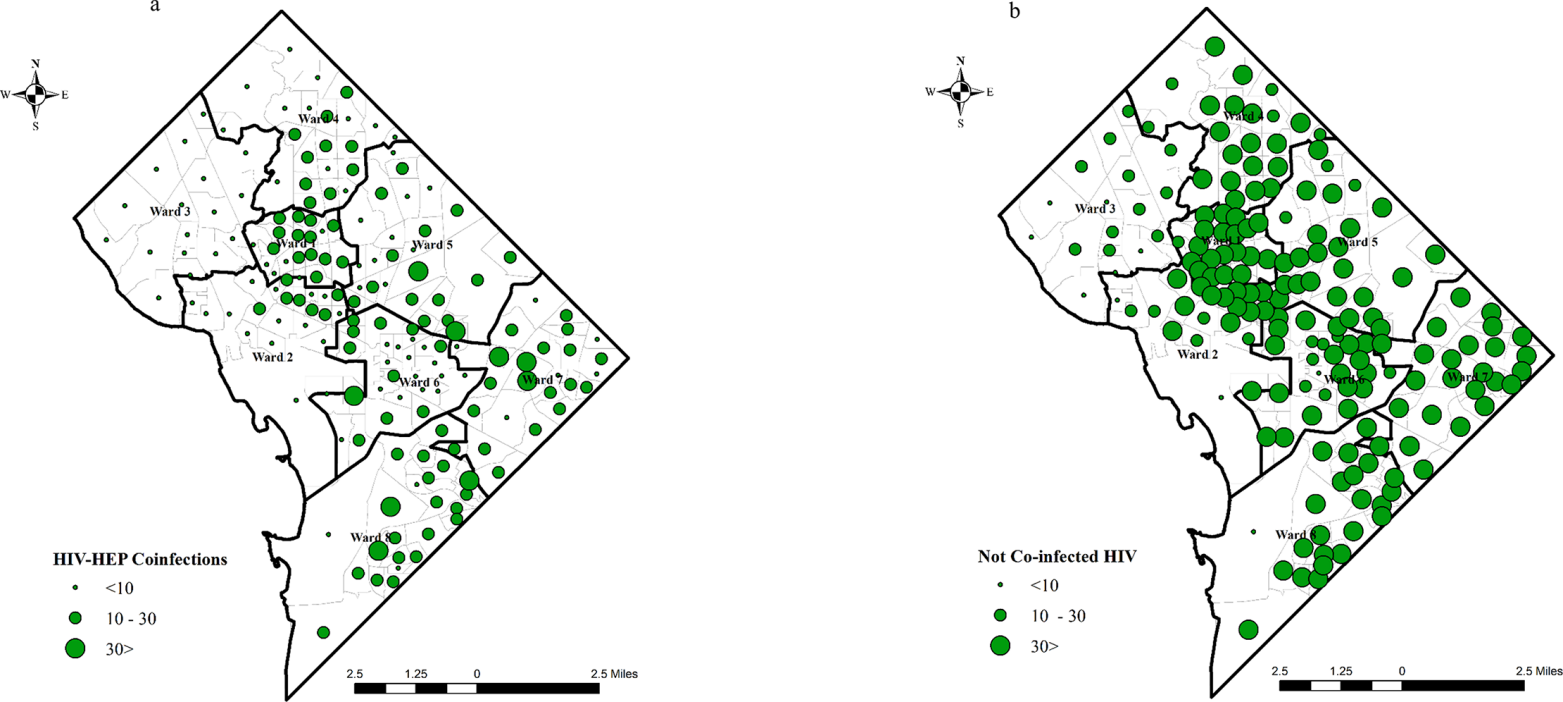
1a and 1b. Distribution of co-infected and not co-infections cases in District of Columbia.

Fig 1A and 1B shows the distribution of co-infected cases and not co-infected cases. The co-infected population show the lowest aggregated cases in wards 2 and 3. The not co-infected aggregated cases are higher in Central DC as well as in wards 7 and 8.

The HIV prevalence (per 10,000) in DC by was mapped (S2 Fig). The showed a higher prevalence of HIV infections in Central DC in Wards 1, spreading to wards 2, 5, 6 and some parts of wards 7 and 8.

The distribution of hepatitis B (HEP B) and C (HEP C) was mapped (S3 Fig). The maps showed similarity in distribution of co-infections. HEP B and C show similar trends in distribution, with 8–14 number of cases as the comparable. As mentioned earlier in the methods section, the similarity in distribution lead us to club the cases together instead of conducting a separate cluster analysis.

**Fig 2:**
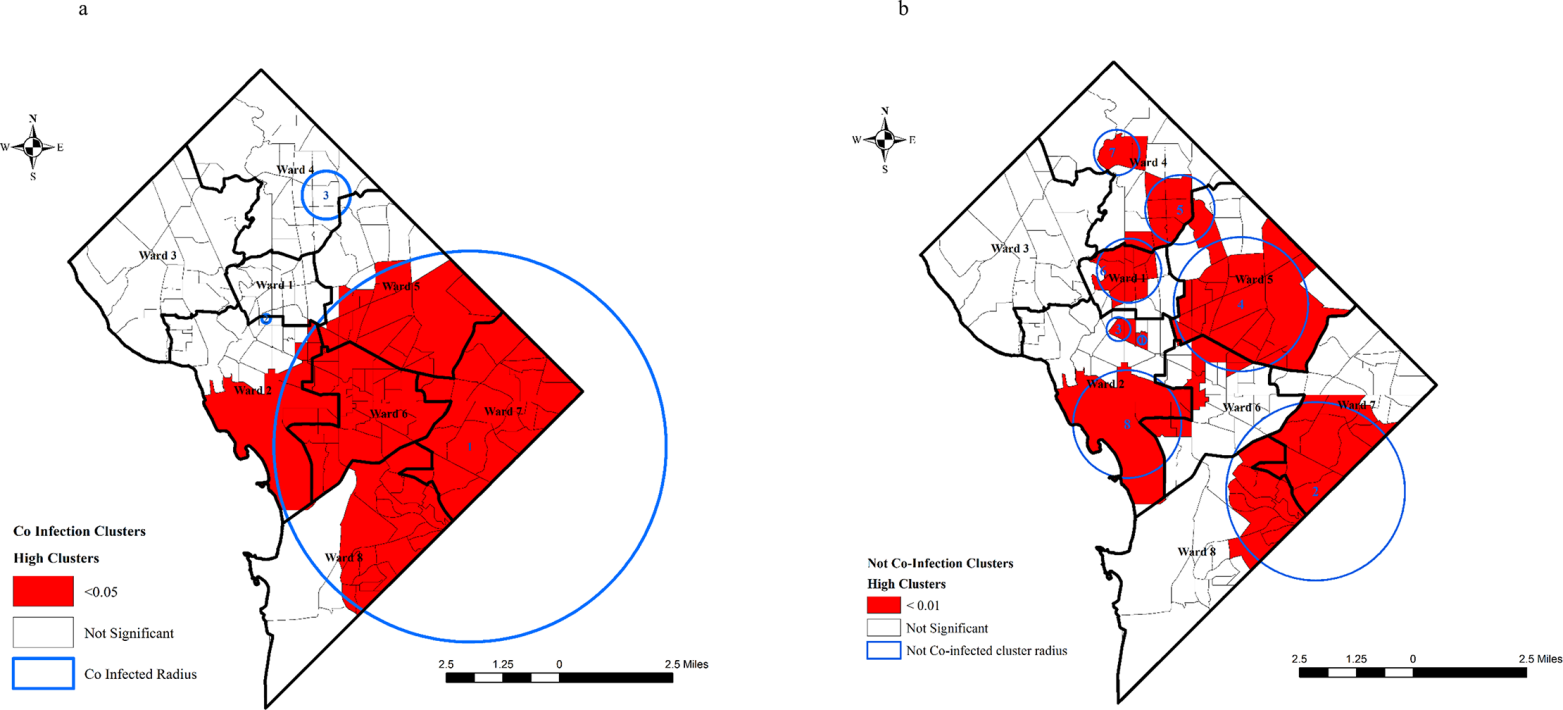
2a and 2b Discrete Poisson clusters of co-infected and not co-infected by tracts. (The numbers in the circles shows cluster number).

The Poisson cluster analysis identified three spatial clusters (Fig 2A) for the co-infected population. For co-infected cluster characteristics (Table 3), cluster 1 covers primarily large parts of southern DC including tracts wards 6 and 7 and large parts of 5, 2 and 8 into Maryland as well with an RR of 2.38 (*p < 0*.*001*) (Fig 2A). Cluster 2 located covering a single tract of DC, at the border of ward 1 and 2 with an RR of 2.16 (*p = 0*.*150*). Cluster 3, located in ward 4 showed a RR of 1.36 (*p = 0*.*980*). Hepatitis B (n = 330) coinfections were highest in cluster 1 followed by cluster 3 (n = 14), while hepatitis C co-infections were highest in cluster 1 (n = 1096) and cluster 3 (n = 36), which demonstrates similarities in their distribution (Table 3).

**Table 3:** HIV-hepatitis B or C co-infection and not co-infected clusters with high rates identified by SaTScan discrete Poisson method, District of Columbia.

**Co-Infection Clusters**					
Cluster numbers	HIV	Observed Cases	Expected cases	Relative Risk	p value
Cluster 1	82	1508	1018	2.38	0
Cluster 2	80	28	13	2.16	0.15
Cluster 3	103	69	51	1.36	0.98
**Not Co-Infected Clusters**					
Cluster 1	151	130	1	128.75	0
Cluster 2	104	1790	979	2	0
Cluster 3	158	275	79	3.55	0
Cluster 4	86	1490	1108	1.4	0
Cluster 5	88	506	365	1.41	0
Cluster 6	93	900	710	1.29	0
Cluster 7	88	158	103	1.55	0.001
Cluster 8	62	150	102	1.48	0.008

The Poisson cluster analysis identified eight clusters (Fig 2B) for the not co-infected population. For not co-infected cluster characteristics (Table 3), cluster 1 had the highest RR of 128.75 (*p < 0*.*001*) concentrated in a single census tract of central DC in ward 2. Cluster 3 adjacent to cluster 1 had an RR of 3.55 (*p < 0*.*001*) also covers single tract in central DC in ward 2. The lowest RR 1.25 (*p < 0*.*001*) was calculated in cluster 6 in ward 1. All of the not co-infected clusters have a *p-value* of less than *0*.*001* (Fig 2B). (Fig 2A and 2B about here).

## Discussion

The largest co-infection cluster was located covering tracts of wards 1, 6 and parts of wards 2, 5 and 8 while not coinfected clusters were spread across the district with smaller radiuses. The co-infected clusters showed higher numbers of IDU as modes of transmission compared to not-coinfected clusters. Though it is also interesting to note that in terms of distribution of HEP B and C District of Columbia do not show any variation.

Though steadily decreasing yet injection drug users (IDU) accounts for 11.7 percent of the living cases of HIV in the District [4]. HIV transmission through IDU disproportionately affects women and African-Americans, and the problem is most common in DC’s most economically disadvantaged areas [26]. It is evident from the analysis (Table 4) that among mode of transmission, IDUs were highest in co-infection cluster 1 (n = 450) followed by MSM (n = 386). Compared to not co-infected clusters where MSM (n = 2,534) and heterosexual contact (HET) (n = 1,532) were primary modes of transmission for all clusters combined. District of Columbia has a local funded needle exchange program which helps keeps new HIV infections low [27] but its impact on hepatitis remains unknown. Past studies have shown that needle exchange programs have also proven successful in preventing hepatitis infections in people who inject drugs [28,29].

**Table 4:** Cluster characteristics by modes of transmission and types of co-infections.

	Co-Infected Clusters				Not Co-Infected Clusters								
Modes of Transmission	Cluster 1	Cluster 2	Cluster 3	Total	Cluster 1	Cluster 2	Cluster 3	Cluster 4	Cluster 5	Cluster 6	Cluster 7	Cluster 8	Total
MSM	386	1	13	400	76	648	218	708	215	539	36	94	2534
IDU	450	0	13	463	9	178	4	104	26	38	5	7	371
MSM/IDU	88	0	4	92	4	45	10	51	11	16	3	5	145
HET	363	0	15	378	22	629	14	394	175	196	82	20	1532
RNI	131	0	5	136	17	261	29	218	73	104	29	23	754
**Types of HEP**													
HEP B	330	1	14	345									
HEP C	1096	0	36	1132									

From the results, we also found that MSM bears the second largest burden of co-infections. MSM and long-term partners of persons with chronic infection have been shown to be at extraordinarily high risk for acquiring Hepatitis and HIV co-infection [30]. CDC funded 1509 program in DC provides comprehensive prevention, care, behavioral health, and social services for MSM of color at risk for and living with HIV infection.

To understand the underlying etiology of the co-infected areas, it is important to recognize the disparity in demographic and economic characteristics. It is also important to evaluate the spatial disparity of socio-economic characteristic of co-infected people in order to prevent co-infections and provide effective care. To gauge we selected four indicators of social-determinants of health, black and white population, poverty and high school drop outs to characterize the clusters also inform population based policies.

**Fig 3:**
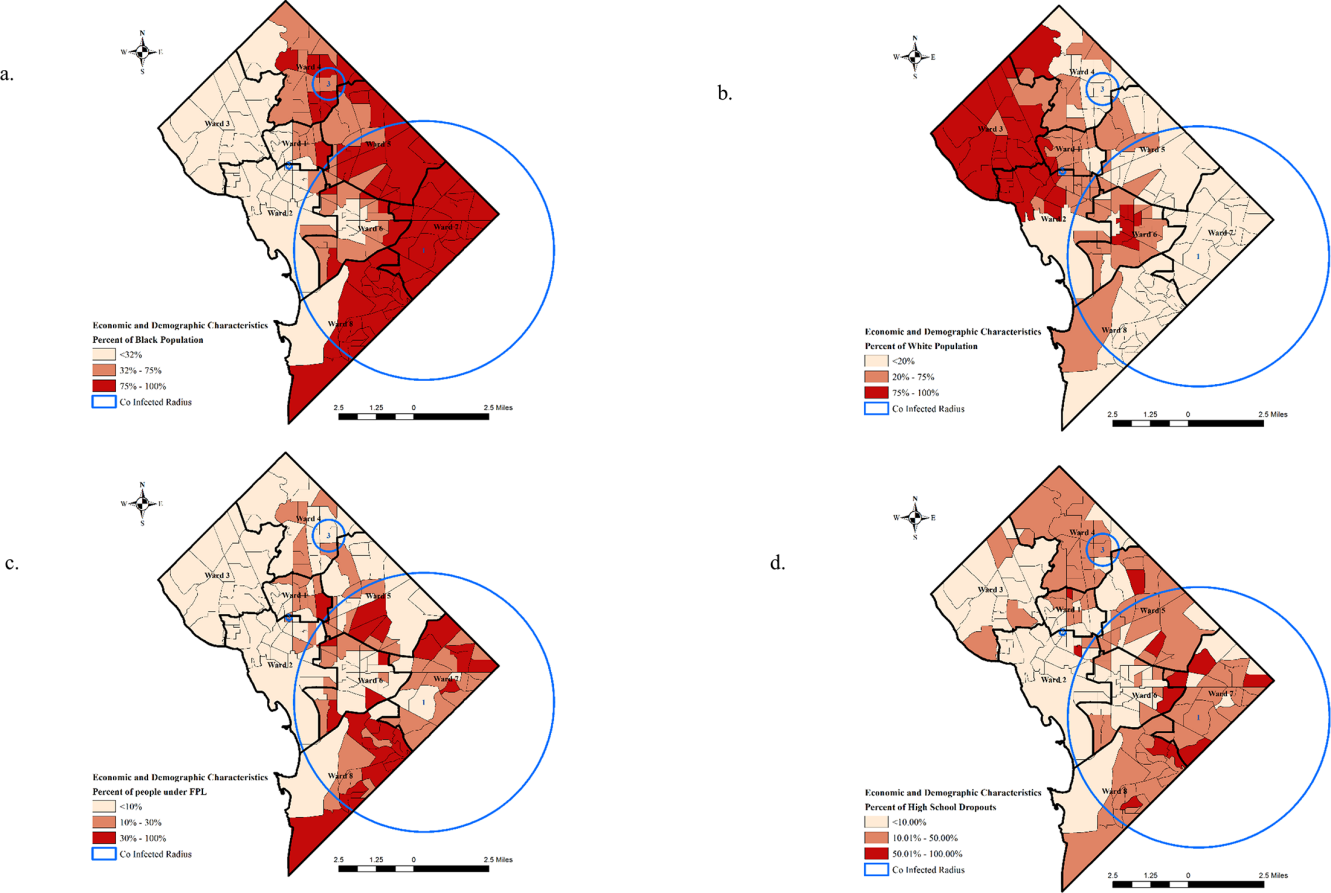
Percentage distribution of (a) black population, (b) white population in the tracts of District of Columbia, (c) people below federal poverty level (FPL) and (d) high school dropouts in the tracts of District of Columbia based on American Community Survey (ACS) in coinfected clusters.

The percentage of black population is higher in the tracts that fall in the co-infected cluster radius (Fig 3A). The co-infected tracts have more than 70 percent of population who are black or African American while less than 5 percent of the population were white (Fig 3B) in clusters 1 and 3. Previous studies have shown that race plays an essential part in HIV-Hepatitis co-infection risk [31]. Racial residential segregation leads to racial disparities in health outcomes, clusters of HIV-Hepatitis co-infections were no different. African-American experience considerably higher levels of residential segregation [32,33]. Despite the lack of supportive legal statutes, the scale of residential segregation remains exceptionally high for mainly African Americans in the United States [34]. Though DC has a higher percentage of African American population, but the residential segregation calls for a change in policies that will have an overall impact on the health of people living in areas of high co-infections.

More than 28 percent of the population lives below federal poverty level (FPL) in the tracts that were located in the co-infected clusters (Fig 3B). Analyzing disease surveillance data according to area-based poverty measures helps outline residents who are at increased risk for a disease, a vital step toward recognizing these disparities and targeting prevention measures [35]. Poverty not only impedes diagnosis but also reduces access to treatment [36,37] and the impact HIV epidemic has been higher among the economically underprivileged in urban areas [38].

School education have been one of the strongest predictors of health outcomes. In the past research has shown compelling evidence which shows that education has an impact peoples’ earning, concurrently it is also being suggested that education wields the strongest impact on health [39–41]. High school completion is a suitable measure of educational achievement since its impact on health is well studied, and it is broadly known as the least entry condition for higher education and well-paid employment [40]. The results from the analysis show that a large number of tracts in the high-risk co-infected clusters have more than 10 percent who are high school drop outs, while few tracts demonstrated more than 50 percent of the population who are high school dropouts.

Economic and social conditions define the extent to which a person possesses the physical, social and personal resources to identify and achieve health [42]. Examination of the spatial patterns of HIV-Hepatitis co-infection could provide new understandings about the drivers of transmission. This understanding is essential as hepatitis can be cured, but may be cost‐prohibitive which makes treatment inaccessible [43]. The inaccessibility may stem from various factors such as racial residential segregation, poverty and lack of education as discussed in the analysis. The results would probably provide direction to policymakers to ascertain the areas for optimum prevention and intervention programs.

Apart from the disparity in few of the socio-economic variables mentioned in the analysis another potential explanation of these geographic difference of the results could be attributed to the absence of proper prevention funding and lack of active hepatitis surveillance. This makes it difficult for the local health departments to provide care as well as monitor who need hepatitis treatment even though the treatment is available. Though World Health Organization (WHO) stresses expansion in the surveillance of Hepatitis and HIV is vital to aid outline the epidemiology of coinfection and advise suitable strategies for testing, prevention, care and treatment to those in need [44].

To summarize this analysis identified the areas of high HIV-Hepatitis co-infections in DC. The clusters radius expands into the neighboring state of Maryland as well. As of 2016, 10 percent of IDU based HIV infections who were diagnosed in DC have out-migrated [4]. It is imperative to understand the burden of co-infection among these out-migrated population. Based on reports published by the Office of Planning, Government of District of Columbia, Maryland remains the top receiver of out-migration from DC[45]. Collaborative efforts between the health departments can have a significant effect on the burden of coinfections in the region. Further, HIV and hepatitis programs which will cater to Black MSM and IDU are recommended for the high risk clusters.

Further to enhance prevention and reduce the risk of new co-infections it is important to have active hepatitis surveillance which would help identify the epidemiology of the co-infection which in turn would further assist prevention and intervention strategies.

## Conclusion

In conclusion, this analysis identified significant clusters of co-infection in tracts that can be considered high risk because of underlying socioeconomic characteristics of the population. The analysis is a significant contribution which has the potential to drive policies when hepatitis does not have any funding for active surveillance and the health departments are directed to care for patients with medicines which are expensive. The results of this analysis would assist the DOH to target interventions such as medical assistance, viral suppression measures as well as suitable prevention methods.

Spatial cluster analysis functions as an essential instrument to outline infectious disease clusters, which could be neglected by other statistical methods that ignore geographical dimensions[46]. Also, adequate distribution of resources to these clusters can be considered as a means to attain optimum benefit from any measures that can prevent the spread of hepatitis infection among the high-risk population of HIV-infected individuals. The analysis is limited by space and the time dimension that needs to studied as well, particularly concurrent with the US drug approval timeline. DOH is currently conducting an analysis that uses impact of time and prevention intervention strategies on the HIV and hepatitis in DC. Future research will also focus on identifying risk factors that may be associated with clustering of HIV-HBV/HCV coinfection in these tracts as well as adaptable factors that may help to prevent these infections.

## Supporting information

S1 Fig The boundary map of district of columbia, delineating wards, neighborhoods and census tracts. The blue lines demarcate 179 census tracts and the black lines show the 8 wards of DC.
(TIF)

S2 Fig HIV prevalence and prevalence in Wards, 2002–2016. (TIF)

S3 Fig Aggregated distribution of Hepatitis B and C distribution in the wards of DC. The cases were aggregated by census tracts and overlaid with the ward map.
(TIF)
