# Supplementary figures and images for: Detecting spatial clusters of HIV and hepatitis coinfections
Source: PLoS One. 2018 Sep 18;13(9):e0203674. doi: 10.1371/journal.pone.0203674 (PMC6143237; doi:10.1371/journal.pone.0203674)

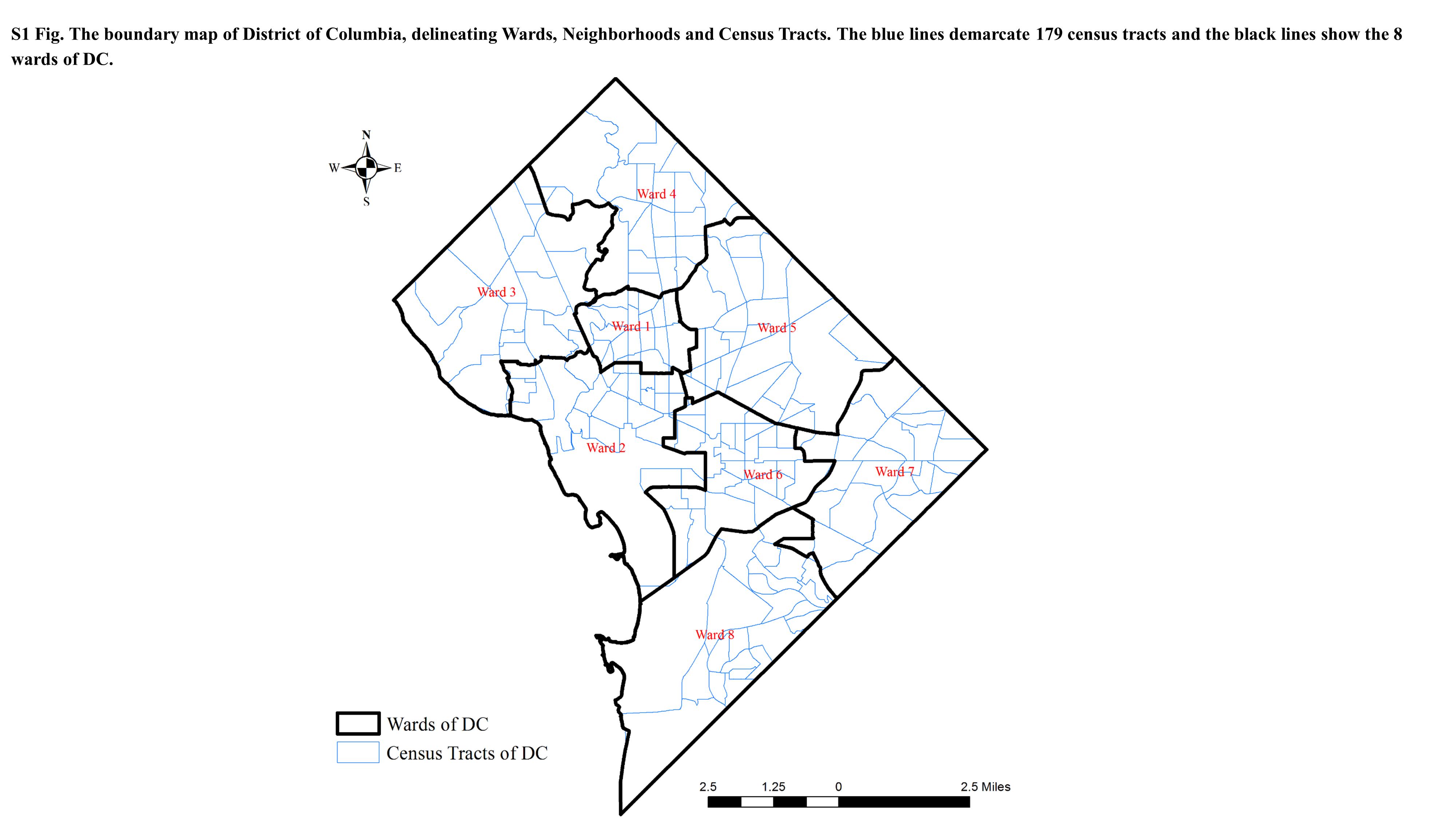

Supplement: S1 Fig — The blue lines demarcate 179 census tracts and the black lines show the 8 wards of DC. (TIF) [file pone.0203674.s001.tif]

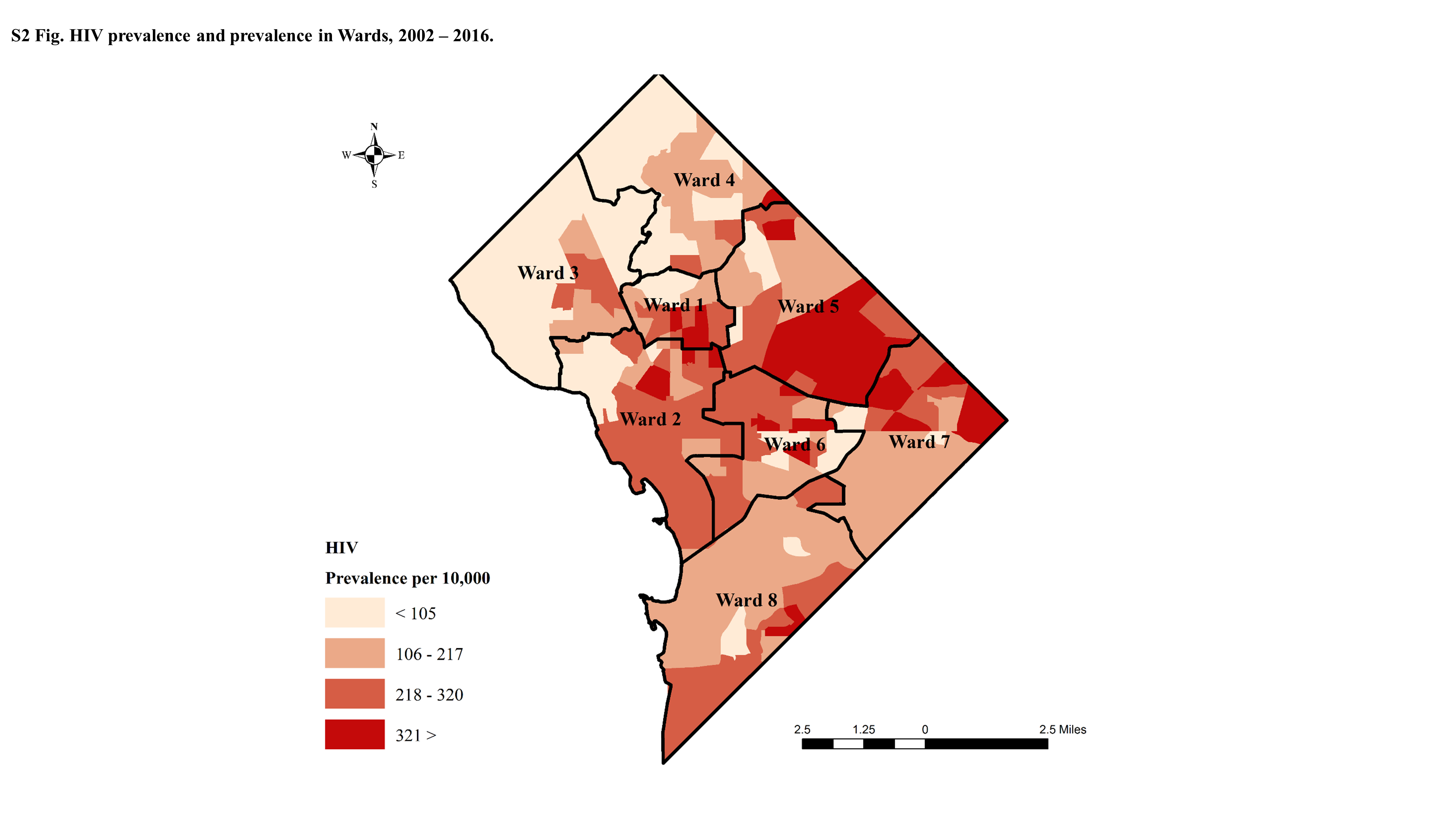

Supplement: S2 Fig — (TIF) [file pone.0203674.s002.tif]

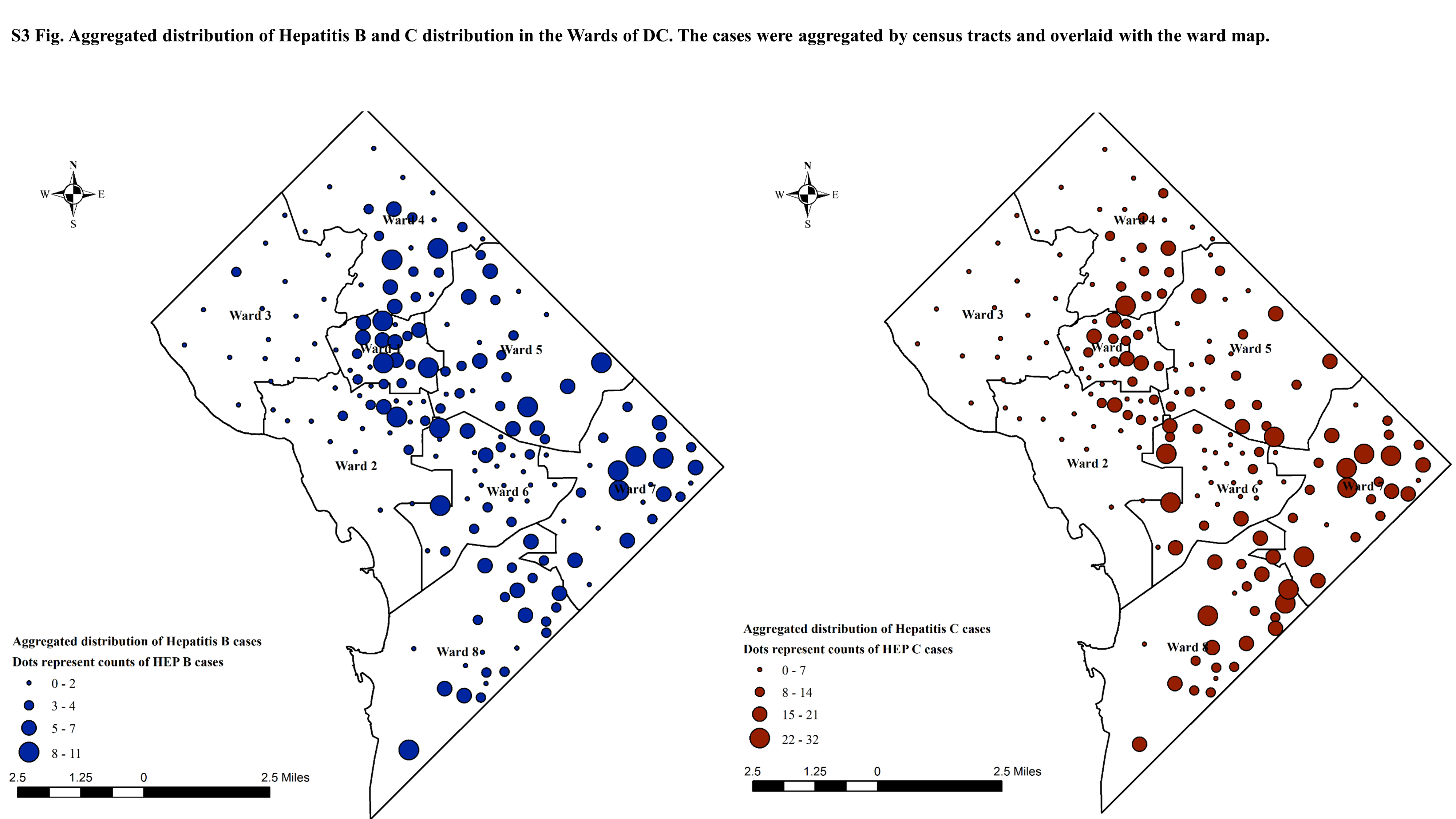

Supplement: S3 Fig — The cases were aggregated by census tracts and overlaid with the ward map. (TIF) [file pone.0203674.s003.tif]
